# Microbial Contamination in Next Generation Sequencing: Implications for Sequence-Based Analysis of Clinical Samples

**DOI:** 10.1371/journal.ppat.1004437

**Published:** 2014-11-20

**Authors:** Michael J. Strong, Guorong Xu, Lisa Morici, Sandra Splinter Bon-Durant, Melody Baddoo, Zhen Lin, Claire Fewell, Christopher M. Taylor, Erik K. Flemington

**Affiliations:** 1 Department of Pathology, Tulane University, New Orleans, Louisiana, United States of America; 2 Tulane Cancer Center, Tulane University, New Orleans, Louisiana, United States of America; 3 Department of Genomic Medicine, University of California, San Diego, California, United States of America; 4 Department of Microbiology and Immunology, Tulane University, New Orleans, Louisiana, United States of America; 5 University of Wisconsin Biotechnology Center, University of Wisconsin, Madison, Wisconsin, United States of America; 6 Department of Microbiology, Immunology & Parasitology, Louisiana State University School of Medicine, New Orleans, Louisiana, United States of America; 7 Research Institute for Children, Children's Hospital of New Orleans, New Orleans, Louisiana, United States of America; The Fox Chase Cancer Center, United States of America

## Abstract

The high level of accuracy and sensitivity of next generation sequencing for quantifying genetic material across organismal boundaries gives it tremendous potential for pathogen discovery and diagnosis in human disease. Despite this promise, substantial bacterial contamination is routinely found in existing human-derived RNA-seq datasets that likely arises from environmental sources. This raises the need for stringent sequencing and analysis protocols for studies investigating sequence-based microbial signatures in clinical samples.

The advent of next generation sequencing (NGS) technology has revolutionized the way pathogens can be detected, studied, and discovered. NGS lends itself to highly sensitive, relatively unbiased, global assessments of all known exogenous agents within biological specimens, including human biopsies. Several laboratories, including ours, have successfully utilized NGS for the discovery and investigation of exogenous agents associated with several human diseases, such as the recent association of fusiform bacteria with colorectal carcinoma [1]–[7]. NGS-based approaches also have great potential in the clinic for the diagnosis of symptomatic infections. Early studies examined microbial sequence-based signatures in feces from patients with diarrheal disease and in urine from patients suspected of having a urinary tract infection to identify the infectious cause [8], [9]. In a recent case report, NGS was used to diagnose a patient with a rare but treatable bacterial meningoencephalitis caused by leptospirosis, a condition which was undetectable using current clinical assays [10].

With the great potential of NGS for pathogen analysis of clinical samples, opportunities are being discussed and bioinformatics challenges are being addressed [11], [12]. While the discussion of opportunities and bioinformatics challenges is highly appropriate, data reliability and contamination, issues that are especially relevant to the inquisitive nature of this application, are scarcely discussed. For some of the current mainstream applications of NGS, such as host transcriptome quantification, reproducibility studies across sequencing centers are being performed to assess data veracity [13]. At a minimum, data reliability in pathogen sleuthing also needs to be thoroughly tested and analyzed, and potential obstacles need to be addressed.

## Bacterial Reads in Multiple Human-Derived RNA-seq Datasets

During the course of DNA and RNA sequencing experiments performed in our laboratory over the past several years, we invariably noted surprising levels of bacterial reads whether the genetic material was derived from human clinical specimens, tissue culture cells, or animal tissues. The extent and pervasiveness of this observation led us to investigate this issue using data from a variety of other publically available data sources. As a first line of investigation, we downloaded RNA-seq datasets from 93 invasive breast carcinomas [14], 15 kidney renal papillary cell carcinomas, 18 lung adenocarcinomas [15], 38 lung squamous cell carcinomas, and 50 rectum adenocarcinomas [16] from The Cancer Genome Atlas (TCGA) cohort (originally made available from the database of Genotypes and Phenotypes [dbGaP] [phs000178]). Colorectal carcinoma (CRC) RNA-seq datasets from Castellarin et al. were downloaded from the National Center for Biotechnology Information (NCBI) Sequence Read Archive (accession number SRP007584) [2]. We also downloaded RNA-seq datasets from normal human tissue samples from the Illumina Human Body Map 2.0 project (from the NCBI Gene Expression Omnibus (GEO) database [GEO accession number: GSE30611]). In total, we analyzed RNA-seq datasets from 244 different specimens from different sources and from different specimen types (Table S1). Ten specimens were identified as outliers based on poor alignment percentages to the human genome (using the robust regression and outlier removal (ROUT) method in GraphPad Prism [version 6 Mac, www.graphpad.com]) and excluded from the analysis.

Metatranscriptome analysis was performed using our computational pathogen detection pipeline, RNA CoMPASS [17]. Briefly, reads ranging from 42–101 nucleotides long were aligned to the human reference genome, hg19 (UCSC), plus a splice junction database (which was generated using the make transcriptome application from Useq [18]; splice junction radius set to the read length minus 4), and abundant sequences (which include sequence adapters, mitochondrial, ribosomal, enterobacteria phage phiX174, poly-A, and poly-C sequences) using Novoalign V3 (www.novocraft.com [-o SAM, default options]). Nonmapped reads were isolated and subjected to consecutive BLAST V2.2.28 searches against the Human RefSeq RNA database and then to the NCBI nucleotide (nt) database to identify reads corresponding to known exogenous organisms [19], [20]. Results from the nt BLAST searches were filtered to eliminate matches with an E-value greater than 10e-6. The results were then fed into MEGAN 4 V4 [21] for visualization of taxonomic classifications.

RNA CoMPASS analysis revealed fairly extensive levels of bacterial reads across all RNA-seq studies analyzed, with average numbers ranging from 1,406 reads per million human mapped reads (RPMHs) in the TCGA datasets to 11,106 RPMHs in the normal tissue from the CRC dataset (Table 1 and Figure S1). Despite the widespread presence of bacteria across groups, different taxa displayed substantial heterogeneity across studies with high levels of *Paracoccus denitrificans* SD1 in the TCGA and BodyMap datasets but not in the CRC dataset, and *Pseudomonas* showing generally high levels in the CRC but not the TCGA or BodyMap studies (Table 1 and Figure S2). The substantial bacterial read numbers for each of these diverse datasets suggest a fairly ubiquitous nature to these findings, and taxa-specific differences across centers raises the possibility of multiple center-specific issues.

**Table 1:** Bacterial profile among various human RNA-seq datasets.

	TCGA	BodyMap	CRC	
			Normal	Tumor
**Human Reads**	773,345±6,104	883,349±3,309	757,775±8,420	757,466±8,640
**Bacterial Reads**	1,406.0±100	1,789.0±242	11,106.0±3,430	9,517.0±3,489
*Acinetobacter*	1.1±0.1	1.3±0.2	4.2±1.2	7.8±1.8
*Fusobacterium*	6.4±2.6	0.0±0.0	53.0±29.0	861.0±491
*Paracoccus denitrificans SD1*	396.0±35	859.0±201	1.6±0.7	1.1±0.63
*Propionibacterium acnes*	16.0±3.9	14.0±3.4	164.0±22	360.0±69
*Pseudomonas*	6.1±0.5	3.0±0.5	2,232.0±393	1,788.0±322
*Enterobacteriaceae*	668.0±94	689.0±166	166.0±75	191.0±74

The average of five RNA-seq datasets (File S1) represent values for TCGA. Similarly, the average of thirteen RNA-seq datasets (File S2) represent values for BodyMap. Colorectal (CRC) RNA-seq datasets were obtained from Castellarin et al. accession number SRP007584 (File S3). All values shown as mean±SEM.

## Identical Cell Lines Analyzed in Separate Studies Show Differences in Bacterial Read Profiles

To shed light on possible contamination sources, we analyzed bacterial reads in cell lines, which we presumed to be free from microbial contamination. RNA-seq data from seven different diffuse large B-cell lymphoma (DLBCL) cell lines that were analyzed independently in the Cancer Genome Characterization Initiative (CGCI) and the Cancer Cell Line Encyclopedia (CCLE) studies were analyzed. CGCI and CCLE RNA-seq datasets were downloaded from dpGaP (phs000235) and the Cancer Genomics Hub (managed by the University of California, Santa Cruz), respectively.

**Figure 1:**
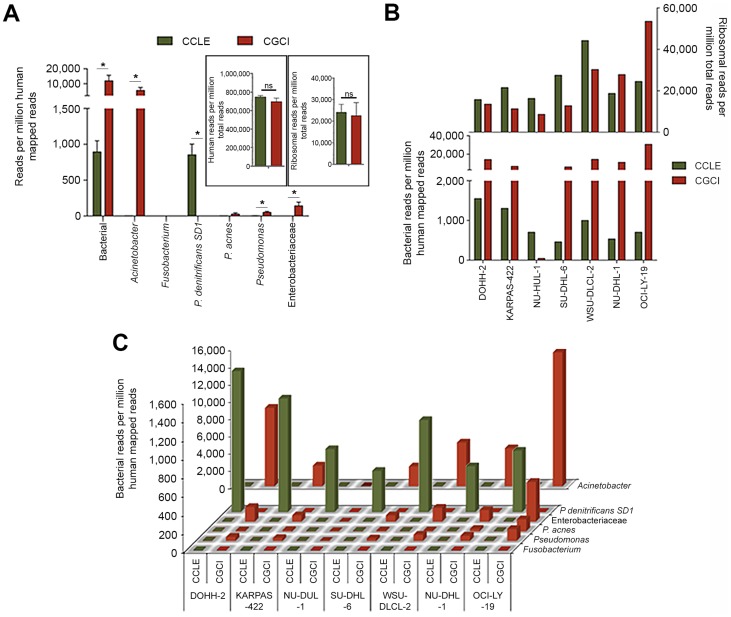
Seven RNA-seq DLBCL cell line datasets sequenced in two different studies (CCLE and CGCI) were analyzed using RNA CoMPASS. (A) Bacterial reads per human mapped reads. For insets, human and ribosomal reads are normalized to total reads. Green columns represent the average RNA-seq reads from the CCLE dataset, while red columns represent the average RNA-seq reads from the CGCI dataset. (B) Mean bacterial RPMHs for each cell line analyzed in the CCLE (green) and CGCI (red) studies with the corresponding mean ribosomal reads (upper graph). (C) Mean RPMHs of various taxa for each cell line analyzed in the CCLE (green) and CGCI (red) studies. *, *p*<0.05.

Based on averaging RPMHs across all cell lines for each study, bacterial reads were found in all datasets, with a considerably greater number in the CGCI study (Figure 1A). *Acinetobacter* was found to contribute to the bulk of bacterial reads in the CGCI data and *P. denitrificans* SD1 made up the majority of bacterial reads in the CCLE study (Figure 1A). Higher bacterial reads were consistently found in all of the CGCI cell lines except for NU-DUL-1 (Figure 1B). In CCLE data, all cell lines were found to be enriched for *P. denitrificans* SD1 reads relative to the CGCI data, whereas the converse was true for *Acinetobacter* (Figure 1C).

The discovery of bacterial reads in cell line data and the finding of different bacterial taxa in data from different sequencing initiatives supports the idea that a good portion of bacterial reads are not derived from the specimens themselves. It is noteworthy that most of these datasets were derived from RNA samples that were polyA selected, a process that selects against most bacterial transcripts (which are typically poorly polyadenylated) [22]–[25]. Contamination that occurs upstream from the polyA selection step, then, is expected to be removed during this purification step. Nevertheless, inefficiencies in polyA selection can result in carry-through of non-polyadenylated bacterial RNAs. If inefficient polyA selection accounted for the majority of bacterial read findings, then we would expect that differences in levels of bacterial reads would relate to differences in polyA selection efficiencies between samples. We assessed polyA selection efficiencies by determining the number of ribosomal RNA reads for each sample, and we found little correlation between the numbers of bacterial reads and the levels of human ribosomal reads (Figure 1A, 1B), supporting the contention that downstream contamination is likely a key source of bacterial reads in these datasets.

## Different Bacterial Read Profiles across Sequencing Centers Using Identical RNA Samples and Library Preparation Kits

**Figure 2:**
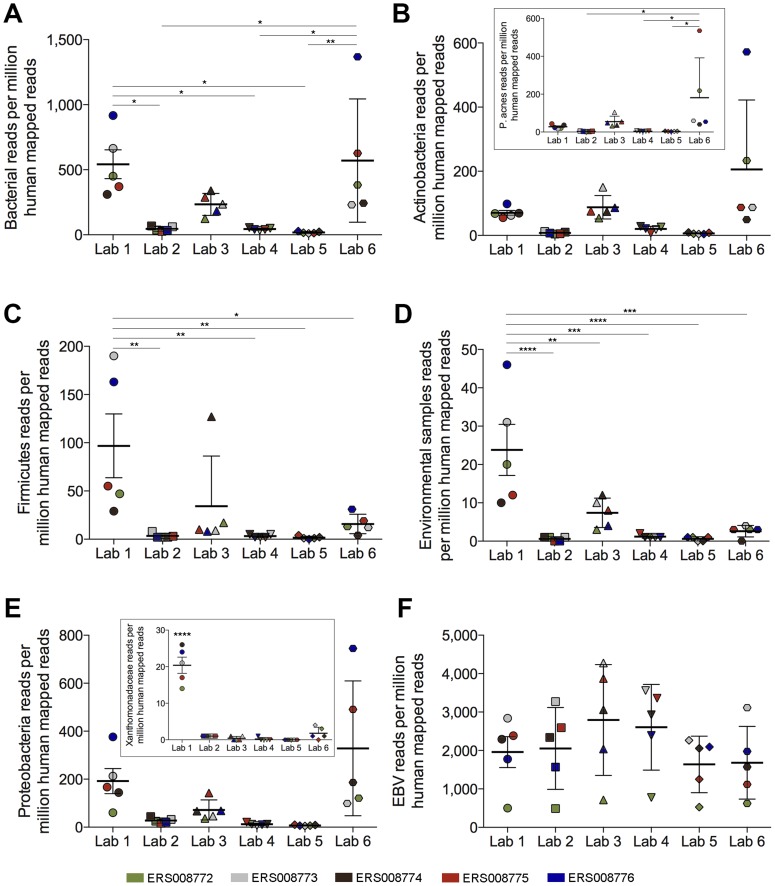
Metatranscriptomic profiles of five RNA sequencing datasets vary across laboratories. Five lymphoblastoid cell line (LCL) RNA-seq datasets, sequenced at six sequencing centers across Europe, were analyzed using RNA CoMPASS. Various classification groups within the bacteria domain for each sample were compared across sequencing centers (A) bacteria, (B) Actinobacteria, (C) Firmicutes, (D) environmental samples, and (E) Proteobacteria. (F) As a control, Epstein-Barr Virus (EBV) read numbers were also analyzed. All reads are normalized to million mapped human reads. The five LCL RNA samples are represented by unique respective colors. *, *P*<0.05; **, *P*<0.01; ***, *P*<0.001; ****, *P*<0.0001.

To more directly address whether downstream contamination can occur, we took advantage of a well-controlled study performed by the Genetic European Variation in Health and Disease (GEUVADIS) consortium [13], [26]. In their pilot study, ERP000177, RNA from five Epstein-Barr virus (EBV)-positive lymphoblastoid cell line (LCL) samples was delivered to seven different sequencing laboratories across Europe to evaluate the reproducibility of sequencing data across various centers. We restricted our analysis to the six laboratories that used Illumina sequencing. For these datasets, library construction at all institutes was performed utilizing identical library preparation kits. Across these labs the level of bacterial RPMHs differed by as much as 30-fold, with Lab 5 showing an average of 18 bacterial RPMHs while Labs 1 and 6 showed an average of 542 and 570 bacterial RPMHs, respectively (Figure 2A). Also noteworthy is the tight clustering of bacterial read numbers in different samples within each lab, suggesting the attribution of bacterial contamination to laboratory practices and/or the environment. Similar to our findings in the DLBCL data, the levels of bacterial reads across centers did not correlate with the levels of human ribosomal RNA contamination, indicating that these differences were not due to polyA-selection disparities (Figures S3–S7). Finally, differences in read levels for different bacterial taxa were found across labs (Figure 2B–2E and Figure S8), including the presence of high Xanthomonadaceae read numbers in all five LCL datasets from Lab 1 (Figure 2E [inset]). In contrast, the read levels for endogenously expressed Epstein-Barr virus transcripts were similar across labs for each LCL (Figure 2F).

## Contamination Levels

Based on our own observations as well as the observations of others [27], [28] we think that bacterial contamination is a relevant issue that needs to be extensively addressed for NGS-based pathogen detection and diagnostic approaches. The amplitudes of contaminating bacterial reads in RNA-seq datasets are likely high enough to be a confounding factor. For example, our analysis of the data from the CRC study that previously reported the association between *Fusobacterium* and CRC [2] showed an average of 861 *Fusobacterium* RPMHs in the tumor samples (Table 1). This is comparable to the levels of *P. denitrificans* SD1 and Enterobacteriaceae found in the Human BodyMap study (859 and 689 RPMHs, respectively) (Table 1). This observation is more notable considering the fact that the data from the BodyMap study was derived from polyA-selected RNAs, whereas the data from the CRC data was generated using ribodepleted RNA (which does not select against bacterial reads).

## Is Contamination a Threat to All Microbial Sequencing Studies?

There are several different approaches to sequencing-based microbial examination that vary based on the starting material; for example, RNA versus DNA, or the investigation of relatively pure microbial samples versus the assessment of heterogeneous samples in which the microbial genetic material is a minor component (such as much of the clinical human tissue-based work). The impact of contamination on data interpretation varies depending on the approach because different methodologies inherently traject different signal-to-noise ratios. Contamination is less relevant for studies utilizing relatively homogeneous microbial communities, but it can be a confounding factor in the assessment of samples in which the predominant genetic material is human (for example, tumor biopsies) or in which the offending microbe is in the minority.

A somewhat less obvious effect on signal to noise ratio is the difference between sequencing RNA versus DNA. Assuming contamination that occurs downstream from the nucleic acid preparation step, there is a larger impact of contaminating microbial DNA on RNA sequencing relative to DNA sequencing approaches. This difference arises due to the inefficiencies in converting RNA to cDNA. Since contaminating DNA does not require this step, the signal-to-noise ratio for RNA-seq is lower than for DNA-seq.

So why not just sequence DNA? There are certainly advantages to sequencing DNA, including its greater stability and the ability to retrieve genetic material from archived samples. Nevertheless, there are also advantages to sequencing RNA for some applications. There is an abundance of publicly available RNA-seq datasets that are potentially useful for future pathogen studies. Another advantage is relevant to the study of human biopsies in which the microbial material is a minor component of the sample. The bacterial-to-human transcriptome size ratio is typically greater than the bacterial-to-human genome size ratio because of the abundance of extra human DNA that is poorly or not expressed. In these cases, it is more cost effective to assess the microbial component through RNA sequencing. An added benefit of RNA-seq for clinical diagnosis is the ability to simultaneously obtain information on expressed pathogenic and resistance markers that can inform treatment options.

In the end, when it makes sense for a particular study, one way to obviate the impact of potential contamination is to use a viable approach that maximizes the signal-to-noise ratio. On the other hand, when methods are required that have inherently lower expected signal-to-noise ratios, alternative approaches are necessary to combat this issue.

## Dealing with Contamination Issues

For some cases, contamination can potentially be dealt with bioinformatically. One approach would be to utilize a repository of common contaminating organisms (although this could potentially result in oversight of a relevant organism that happens to be a common contaminant). Alternatively, for investigations in which negative controls are available (and/or suitable), statistics can be used to prove an association (although contamination could result in the requirement for larger sample sets than would otherwise be necessary to attain statistical significance). Despite the utility of informatics approaches to alleviate contamination issues in some cases, minimizing contamination sources is more cost effective and will minimize the chances of data misinterpretation.

Interestingly, contamination has already had an impact on the very databases that are used for bioinformatics work. Laurence et al. identified *Bradyrhizobium* sequences in assembled genomes in the NCBI Genome database [27]. *Bradyrhizobium* species, along with other microbes, have been reported in ultrapure water systems and may help explain the presence of this microbe in several deposited genome assemblies. Another group found *Leucobacter* sp. sequences in assembled genomes of *Caenorhabditis* sp. [28]. These two cases exemplify the need to sequence contaminant genomes in order to exclude them from the host genome assembly.

Furthermore, in a recent study, Xu et al. discovered National Institutes of Health-Chongqing virus (NIH-CQV) in patients with seronegative hepatitis using NGS [29]. However, two later studies demonstrated that the presence of parvo-like hybrid virus (PHV) and NIH-CQV was actually contamination from silica column-based nucleic acid extraction kits and not bona fide viral infection, indicating that contamination is not restricted to bacterial sequences [30]–[32]. Subsequently, in a follow-up study, the authors of the initial report confirmed that the finding of NIH-CQV in human plasma was due to contamination from the columns [33]. This example underscores the importance of rigorously validating novel pathogen discoveries, and when possible, identifying any potential contaminating sources.

The route between clinical specimen collection to the sequencing reaction is complex with many candidate points of contamination, ranging from specimen contamination in the operating room to storage, sample processing, RNA preparation, library preparation, etc. Another key consideration is the purity of library preparation reagents, many of which (e.g. ligases, polymerases, nucleotides) are purified from bacteria during their manufacture. Depending on the level of purity for these reagents, there is the potential for different levels of bacterial genetic material to be present. Nevertheless, the analysis of the data from the highly controlled GEUVADIS study suggests that laboratory standard operating procedures (SOPs) specific to different sequencing centers is also a critical consideration.

The relative contribution of this panorama of potential contamination sources needs to be parsed in future, expressly designed studies. Until these sources are better understood, we propose the following recommendations:

1. Detection studies, especially with a diagnostic focus, should incorporate stringent SOPs across the entire experimental pipeline from sample collection to sequencing.
2. Highly purified metabolic enzymes and other reagents used in sequence library preparation should be used whenever possible.
3. Standards for the curation of microbial sequences submitted to Genbank and other large-scale databases should be established in order to assess completeness and quality of the assembled genomes.
4. Contamination controls such as mock sequence library preparations should be used to help guide the development of appropriate and effective SOPs for metagenomic and metatranscriptomic studies.

## Supporting Information

Figure S1 **Bacterial reads across RNA-seq datasets.**
(TIFF)

Figure S2 **Various bacterial species reads across RNA-seq datasets.**
(TIFF)

Figure S3 **(A) Human and (B) ribosomal reads per million total reads for ERS008772.**
(TIF)

Figure S4 **(A) Human and (B) ribosomal reads per million total reads for ERS008773.**
(TIF)

Figure S5 **(A) Human and (B) ribosomal reads per million total reads for ERS008774.**
(TIF)

Figure S6 **(A) Human and (B) ribosomal reads per million total reads for ERS008775.**
(TIF)

Figure S7 **(A) Human and (B) ribosomal reads per million total reads for ERS008776.**
(TIF)

Figure S8 **Major bacterial contributors to Proteobacteria taxa.**
(TIFF)

Table S1 **Databases.**
(DOCX)

File S1 **TCGA datasets.**
(XLS)

File S2 **Bodymap datasets.**
(XLS)

File S3 **CRC normal and tumor dataset.**
(XLS)
