# Supplementary figures and images for: Microbial Contamination in Next Generation Sequencing: Implications for Sequence-Based Analysis of Clinical Samples
Source: PLoS Pathog. 2014 Nov 20;10(11):e1004437. doi: 10.1371/journal.ppat.1004437 (PMC4239086; doi:10.1371/journal.ppat.1004437)

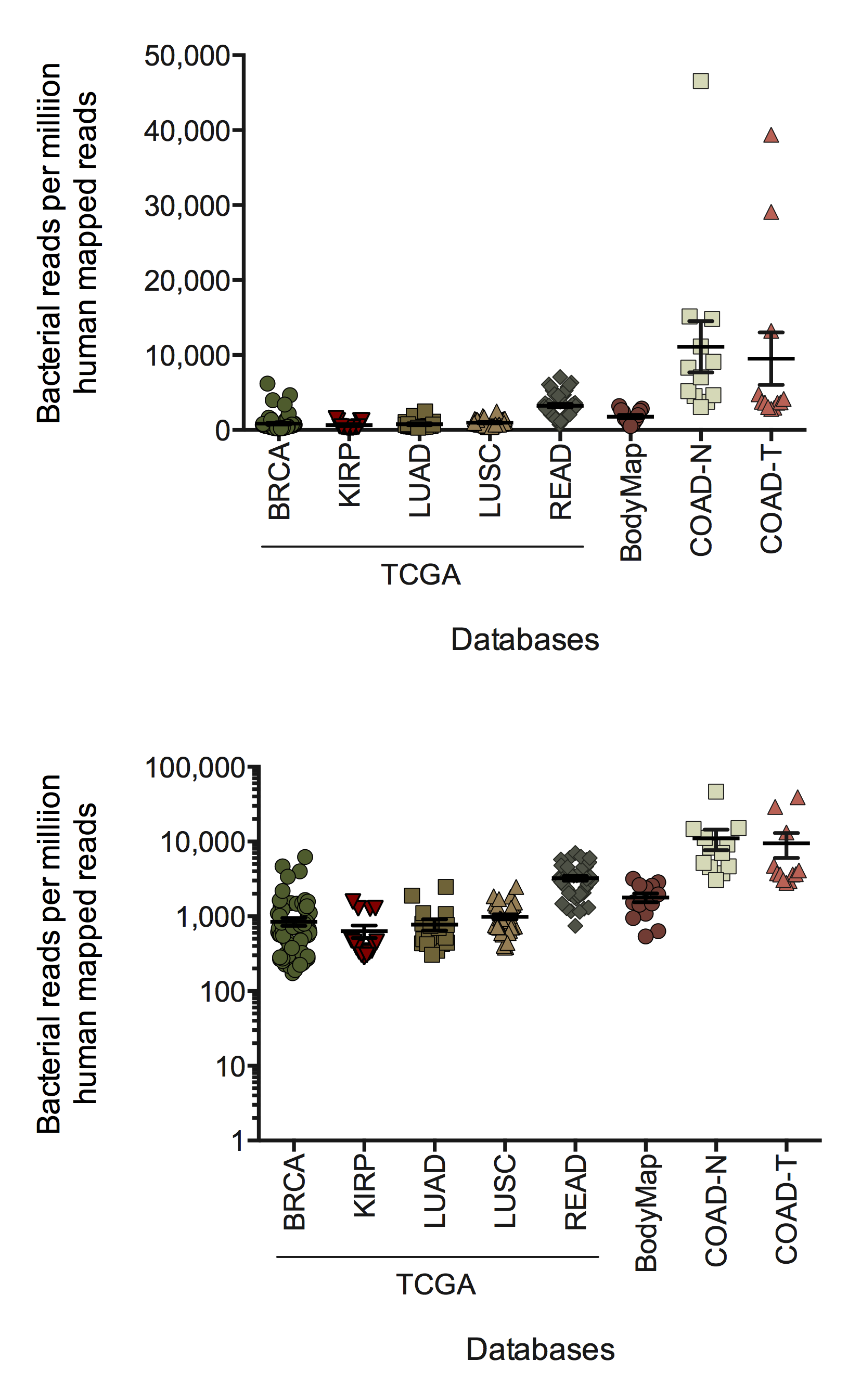

Supplement: Figure S1 — Bacterial reads across RNA-seq datasets. (TIFF) [file ppat.1004437.s001.tiff]

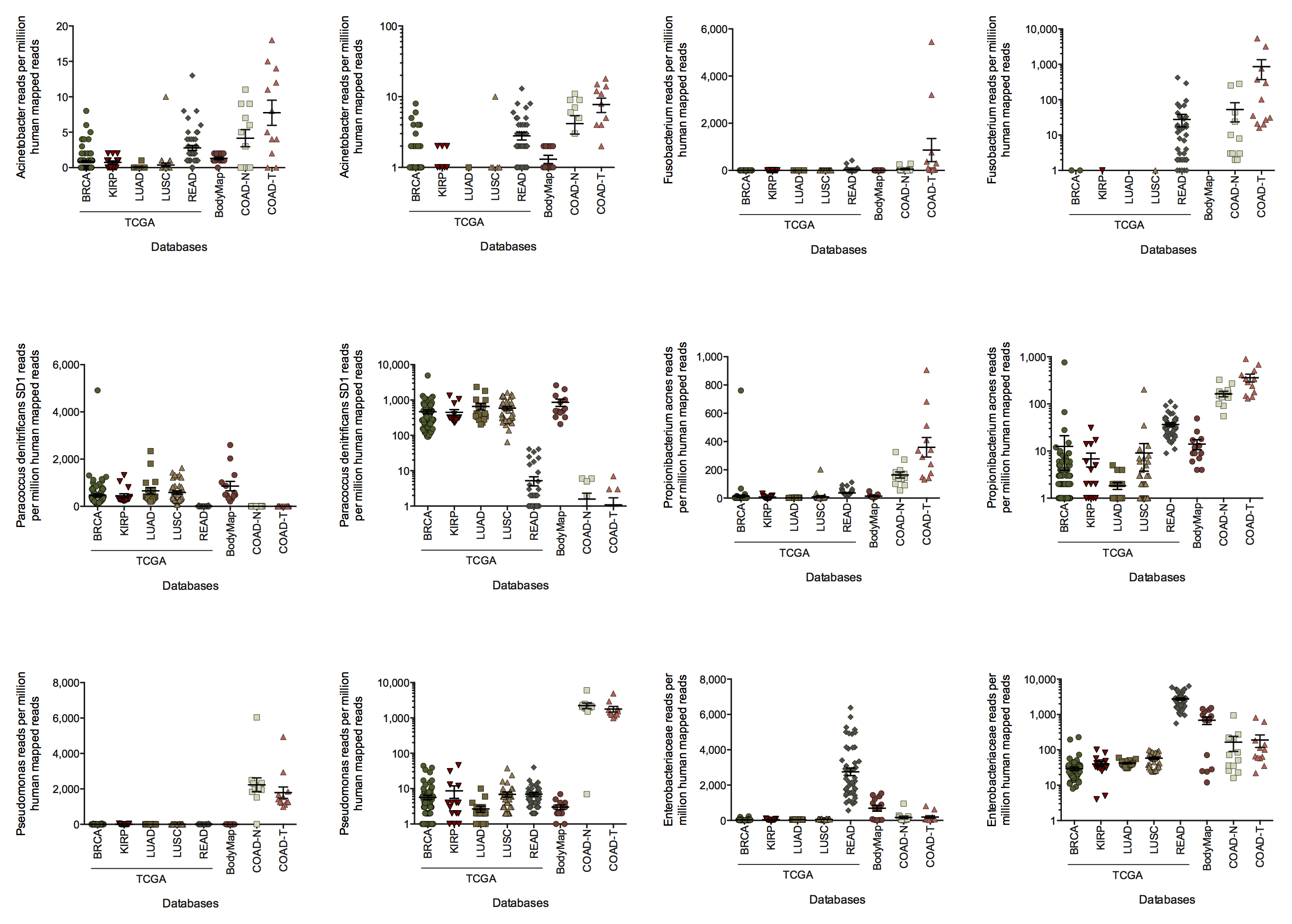

Supplement: Figure S2 — Various bacterial species reads across RNA-seq datasets. (TIFF) [file ppat.1004437.s002.tiff]

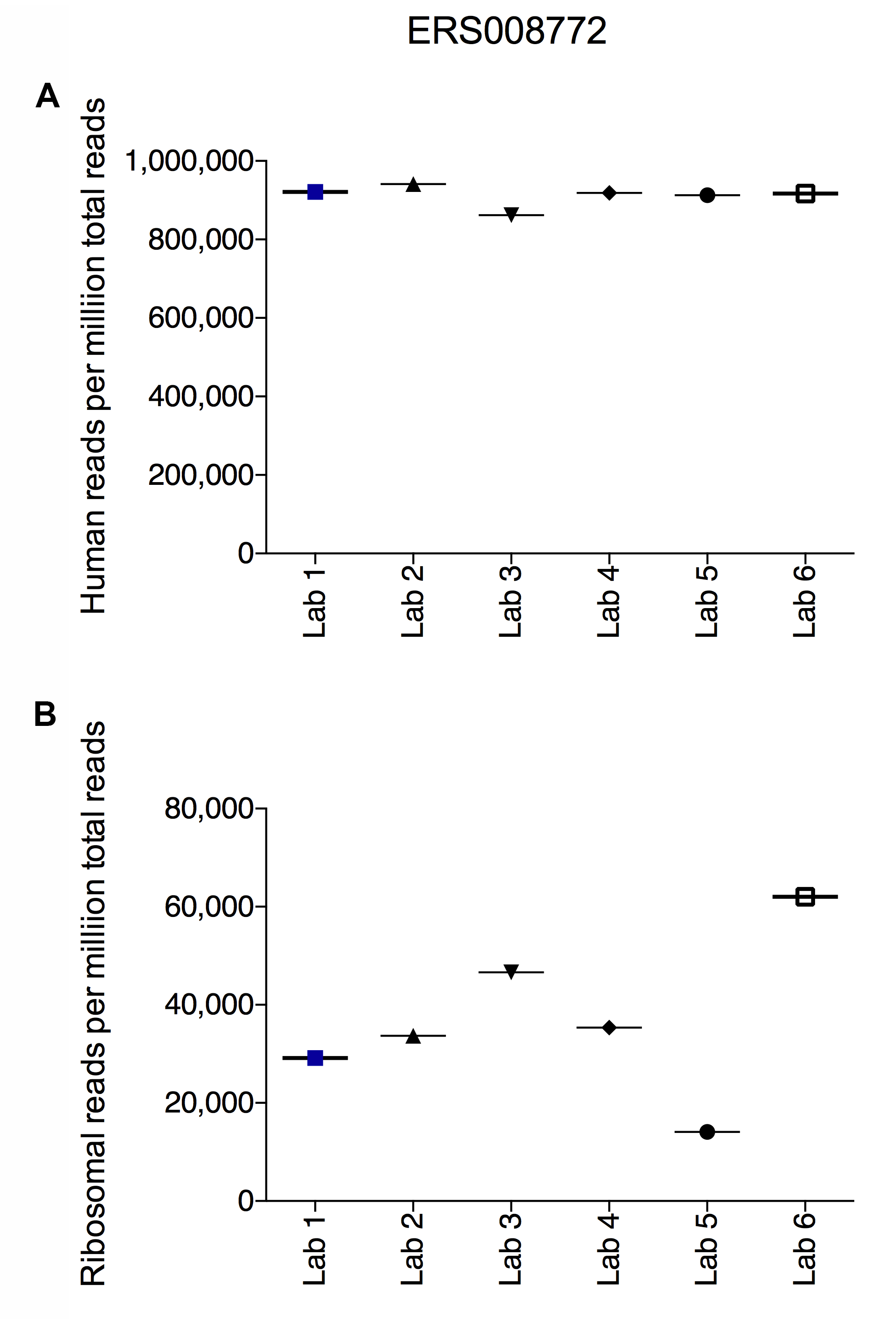

Supplement: Figure S3 — (A) Human and (B) ribosomal reads per million total reads for ERS008772. (TIF) [file ppat.1004437.s003.tif]

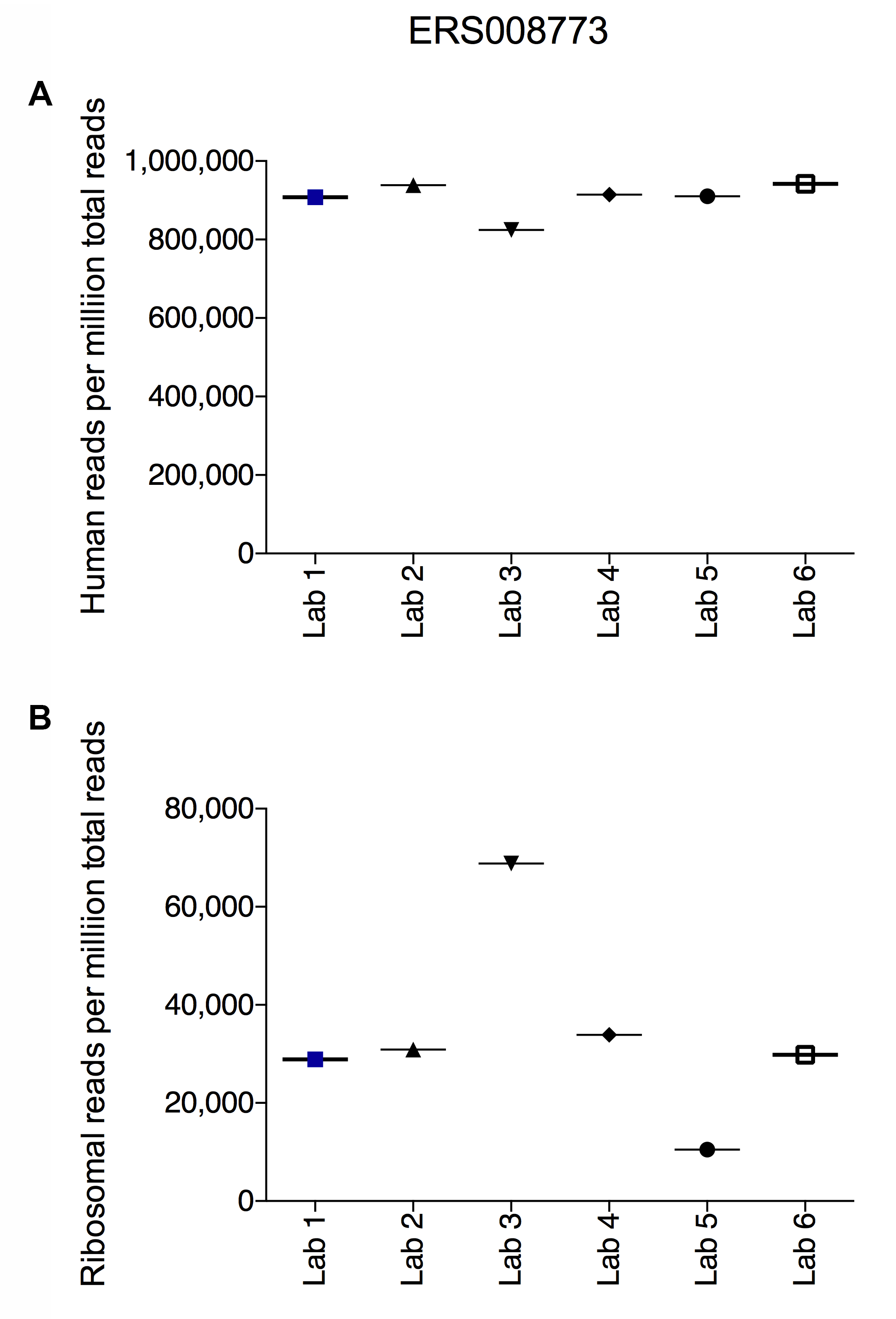

Supplement: Figure S4 — (A) Human and (B) ribosomal reads per million total reads for ERS008773. (TIF) [file ppat.1004437.s004.tif]

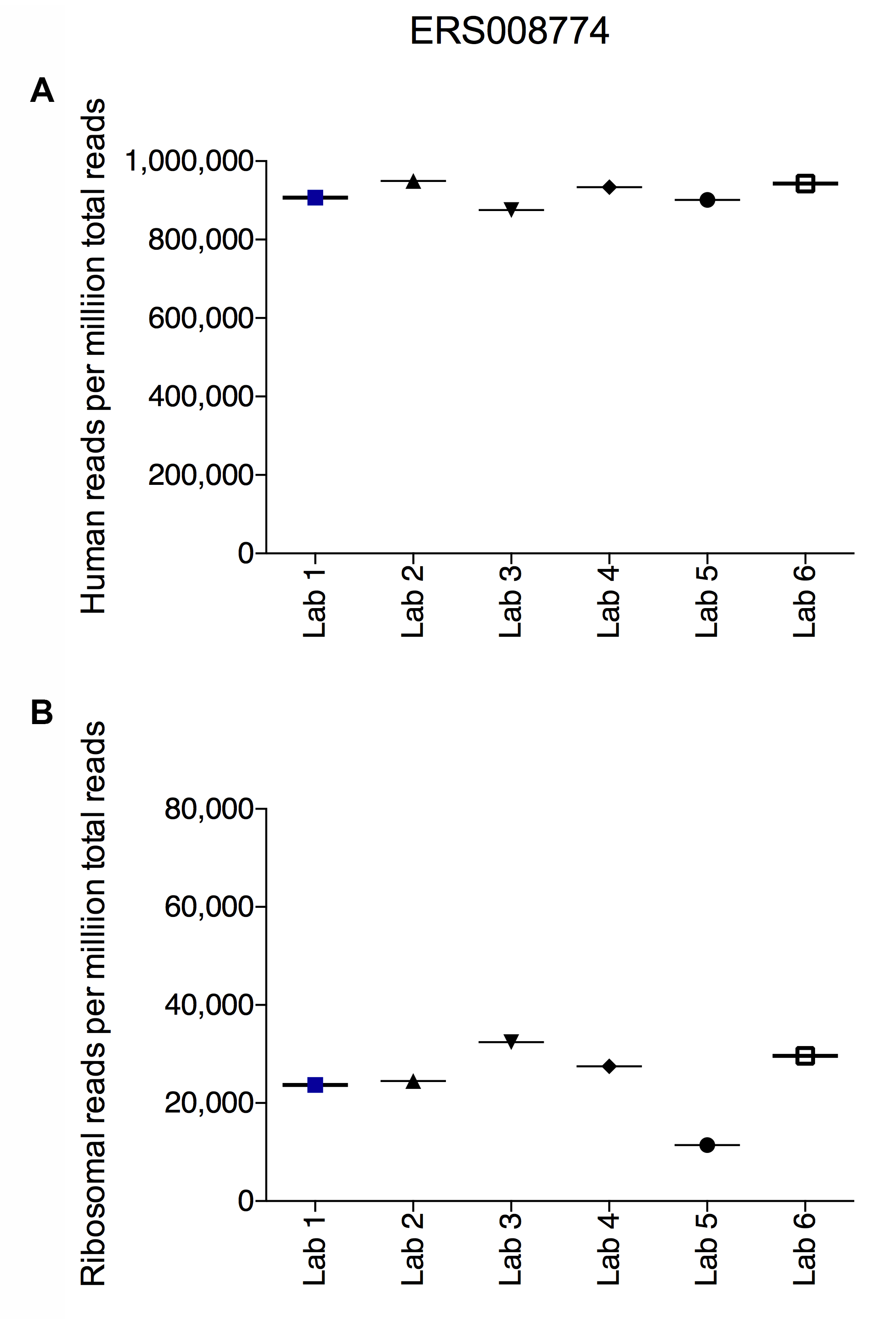

Supplement: Figure S5 — (A) Human and (B) ribosomal reads per million total reads for ERS008774. (TIF) [file ppat.1004437.s005.tif]

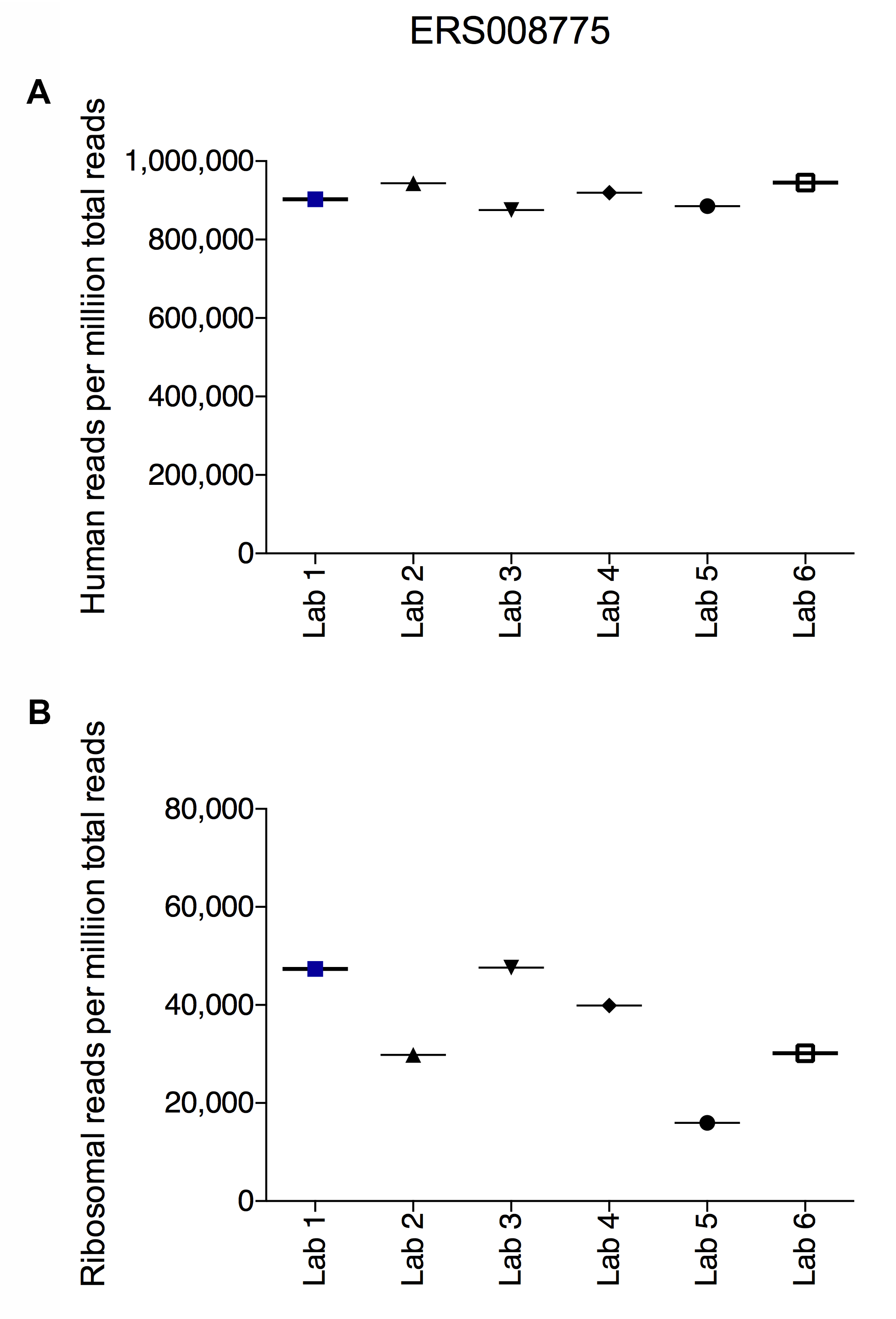

Supplement: Figure S6 — (A) Human and (B) ribosomal reads per million total reads for ERS008775. (TIF) [file ppat.1004437.s006.tif]

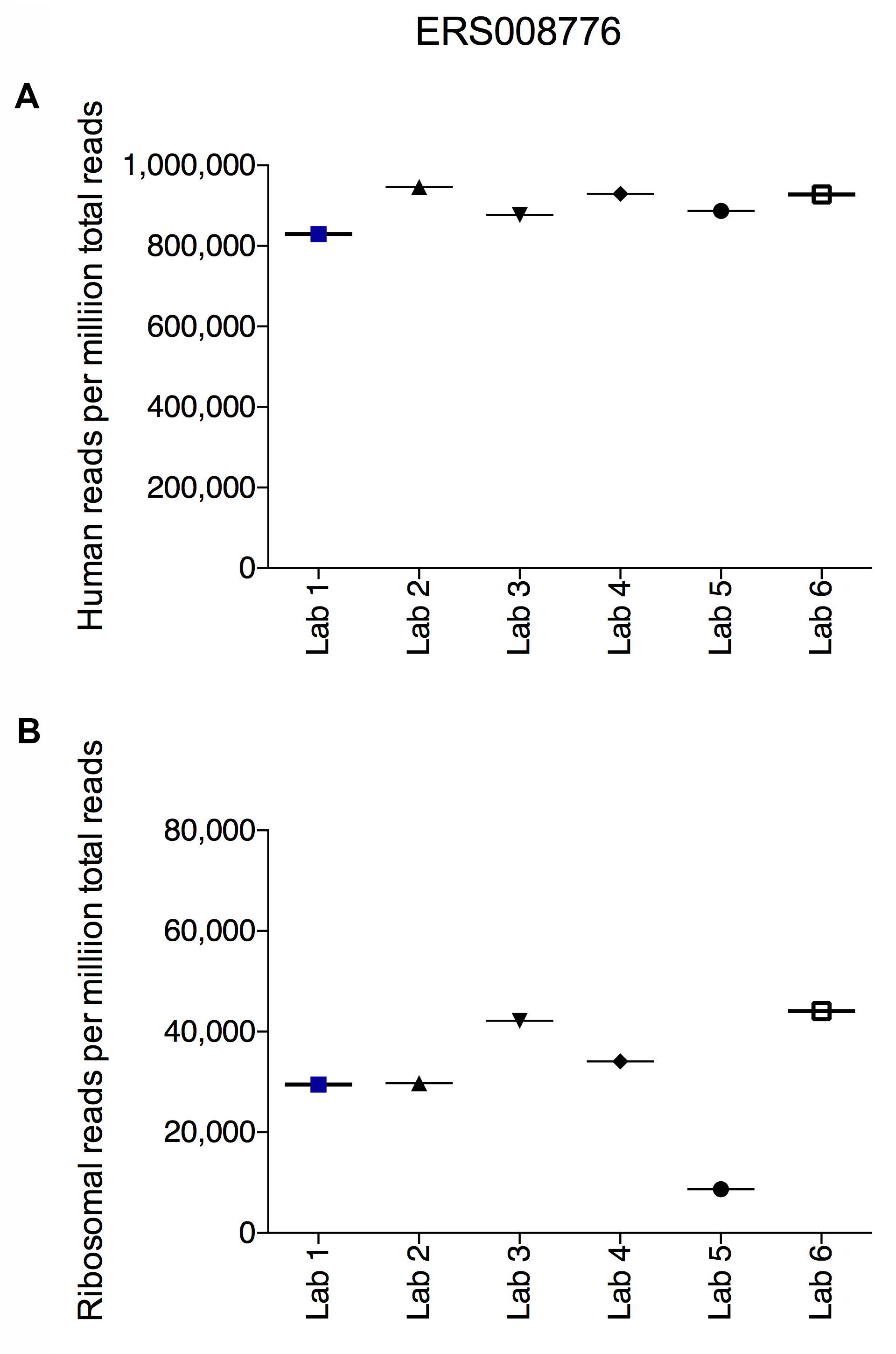

Supplement: Figure S7 — (A) Human and (B) ribosomal reads per million total reads for ERS008776. (TIF) [file ppat.1004437.s007.tif]

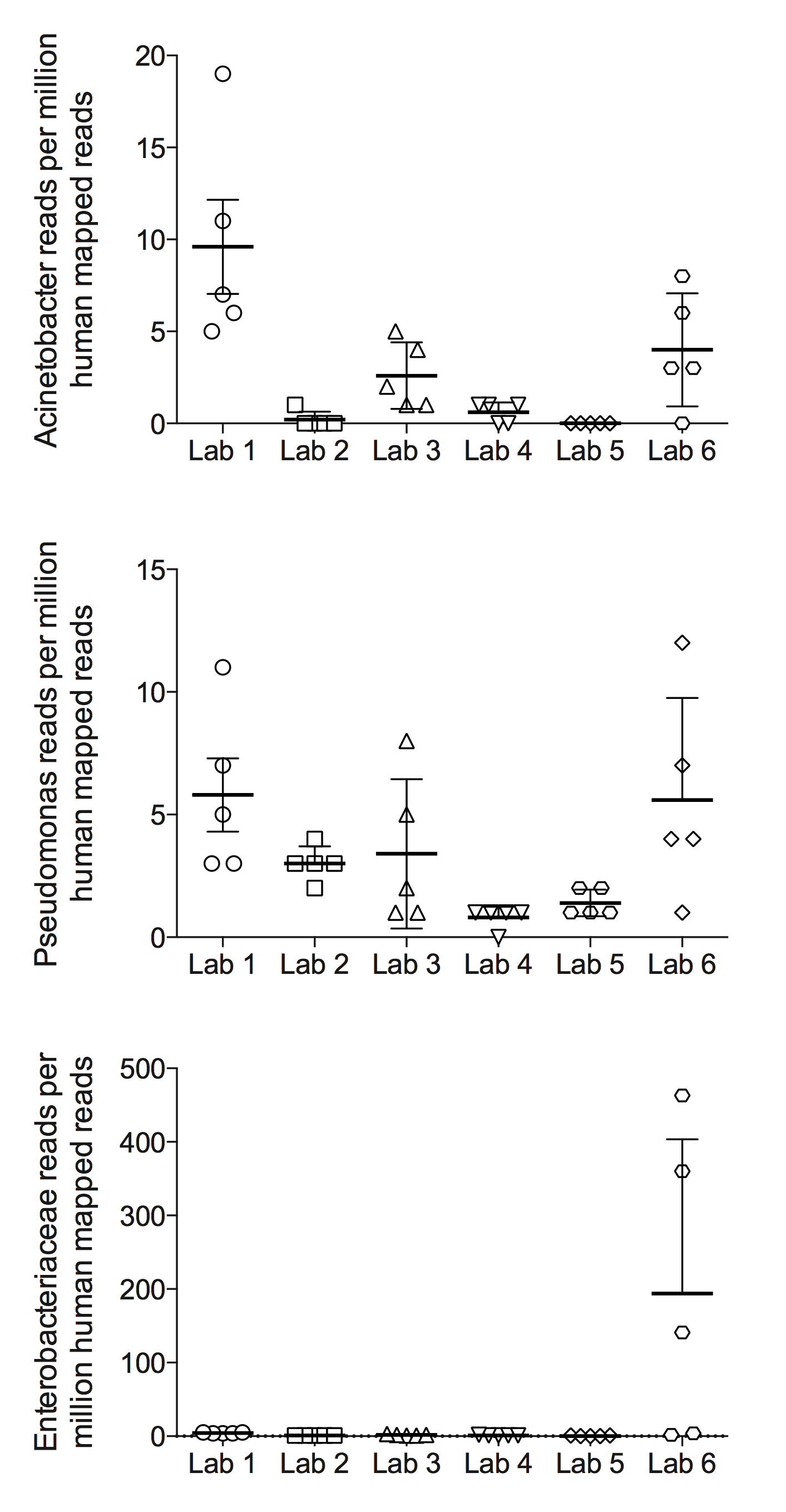

Supplement: Figure S8 — Major bacterial contributors to Proteobacteria taxa. (TIFF) [file ppat.1004437.s008.tiff]
