# Supplementary material for: Microbial Contamination in Next Generation Sequencing: Implications for Sequence-Based Analysis of Clinical Samples
Source: PLoS Pathog. 2014 Nov 20;10(11):e1004437. doi: 10.1371/journal.ppat.1004437 (PMC4239086; doi:10.1371/journal.ppat.1004437)
Supplement: Table S1 — Databases. (DOCX) [file ppat.1004437.s009.docx]

**Table S1. Databases**

| Databases | Number of samples analyzed |
| --- | --- |
|  |  |
| TCGA |  |
| BRCA | 88 |
| KIRP | 15 |
| LUAD | 18 |
| LUSC | 38 |
| READ | 48 |
|  |  |
| BodyMap | 13 |
| Adipose |  |
| Adrenal |  |
| Breast |  |
| Colon |  |
| Heart |  |
| Kidney |  |
| Lymph Node |  |
| Ovary |  |
| Prostate |  |
| Skeletal Muscle |  |
| Thyroid |  |
| Testes |  |
| White Blood Cells |  |
| COAD |  |
| Normal | 12 |
| Tumor | 12 |
